# Bacterial Epibiotic Communities of Ubiquitous and Abundant Marine Diatoms Are Distinct in Short- and Long-Term Associations

**DOI:** 10.3389/fmicb.2018.02879

**Published:** 2018-12-04

**Authors:** Klervi Crenn, Delphine Duffieux, Christian Jeanthon

**Affiliations:** CNRS, Sorbonne Université, Station Biologique de Roscoff, Adaptation et Diversité en Milieu Marin, Roscoff, France

**Keywords:** diversity, heterotrophic bacteria, interactions, diatoms, *Thalassiosira*, *Chaetoceros*, microbiome, Western English Channel

## Abstract

Interactions between phytoplankton and bacteria play a central role in mediating biogeochemical cycling and food web structure in the ocean. The cosmopolitan diatoms *Thalassiosira* and *Chaetoceros* often dominate phytoplankton communities in marine systems. Past studies of diatom-bacterial associations have employed community-level methods and culture-based or natural diatom populations. Although bacterial assemblages attached to individual diatoms represents tight associations little is known on their makeup or interactions. Here, we examined the epibiotic bacteria of 436 *Thalassiosira* and 329 *Chaetoceros* single cells isolated from natural samples and collection cultures, regarded here as short- and long-term associations, respectively. Epibiotic microbiota of single diatom hosts was analyzed by cultivation and by cloning-sequencing of 16S rRNA genes obtained from whole-genome amplification products. The prevalence of epibiotic bacteria was higher in cultures and dependent of the host species. Culture approaches demonstrated that both diatoms carry distinct bacterial communities in short- and long-term associations. Bacterial epibonts, commonly associated with phytoplankton, were repeatedly isolated from cells of diatom collection cultures but were not recovered from environmental cells. Our results suggest that in controlled laboratory culture conditions bacterial–diatom and bacterial–bacterial interactions select for a simplified, but specific, epibiotic microbiota shaped and adapted for long-term associations.

## Introduction

Bacteria eukaryotic microalgae are the major components of the plankton in the upper and ocean layers and their metabolism largely controls pelagic energy flow and nutrient cycling (Falkowski et al., 2008). Determining how they interact is therefore essential to strengthen the understanding of these groups and how they impact marine biogeochemical cycles.

While heterotrophic prokaryotes and phytoplankton are known to interact through complex mechanisms (Azam and Malfatti, 2007), it is expected that they are very closely related in the planktonic environment. The immediate environment of marine phytoplankton cells or phycosphere (Bell and Mitchell, 1972) is considered as physically and chemically distinct from the surrounding seawater, which promote the growth of specific microbial taxa, thus creating a dynamic of interactions which can help to explain the complexity of marine food webs (Seymour et al., 2017 and references therein). The use of rRNA gene sequencing and barcoding approaches allowed establishing links between phytoplankton and bacterial community dynamics in natural communities (Rooney-Varga et al., 2005; Teeling et al., 2012) and culture collections (Schäfer et al., 2002; Jasti et al., 2005; Sapp et al., 2007). These partners often co-occur which lead to beneficial, neutral or parasitic interactions (Amin et al., 2012; Cooper and Smith, 2015; Seymour et al., 2017).

Diatoms are a large component of marine biomass and produce about 25% of the total C fixed on Earth (Nelson et al., 1995; Field et al., 1998). These key ecological players of the modern ocean have been described as the most diverse group of phytoplankton (Armbrust, 2009). Their ecological success is mainly due to their numerous metabolic properties and to their silicified cell wall (Raven and Waite, 2004). Most of their evolutionary adaptations are due to the acquisition of genes from their endosymbiotic ancestors, and by indisputable horizontal gene transfers from marine bacteria, which are rarely documented in other eukaryotic organisms (Armbrust et al., 2004; Bowler et al., 2008). The co-occurrence of bacteria and diatoms in common habitats for more than 200 million years and their intimate associations likely played a major role in the ecological success and species diversification of diatoms (Amin et al., 2012).

Although few reports of diatom–bacterial interactions have used natural diatom populations (Kaczmarska et al., 2005; Rooney-Varga et al., 2005; Amin et al., 2012), most studies were performed using cultures (Grossart, 1999; Schäfer et al., 2002; Grossart et al., 2005; Kaczmarska et al., 2005; Grossart and Simon, 2007; Sapp et al., 2007; Behringer et al., 2018). Consistent associations between specific bacterial and diatom taxa have been found (Schäfer et al., 2002; Amin et al., 2012; Behringer et al., 2018), although other work suggests that the composition of diatom-associated bacterial assemblages shifts over weeks to months in culture (Sapp et al., 2007). Today, however, it remains unclear whether bacteria associated with diatom cells are species-specific (Grossart et al., 2005; Jasti et al., 2005; Rooney-Varga et al., 2005) or determined by bacterial source communities (Kaczmarska et al., 2005; Sapp et al., 2007).

Previous studies on the associations between bacteria and diatoms have mostly considered the bacteria at the population and community levels. The attachment of bacteria to algal cells represents, however, tight associations (Cole, 1982; Grossart et al., 2005). Indeed, Gärdes et al. (2011) demonstrated that attachment of specific bacteria to diatoms *Thalassiosira weissflogii* was required for transparent exopolymer particle formation and aggregation. Surprisingly, little is known regarding the interactions of bacterial assemblages attached to single host cells. The sole exceptions are a microscopy study documenting the abundance and mode of attachment of bacteria attached to individual diatoms (Kaczmarska et al., 2005) and a report evaluating the composition and variability of bacterial assemblages attached to individual diatoms (Baker and Kemp, 2014). More recently, Baker et al. (2016) examined the effect of abiotic and biotic factors on the composition of the attached bacteria associated to a *Chaetoceros* spp. culture.

In this study, our aim was to study tight associations between attached bacteria and diatom partners. For this, we focused on bacteria attached to single cells of the environmentally relevant diatom genera *Thalassiosira* and *Chaetoceros* in natural communities and in culture that were regarded as short- and long-term associations, respectively. These diatoms are ubiquitous and often numerically abundant phytoplankton species in marine systems (Leblanc et al., 2012) and they display the highest species diversity in the pelagic temperate phytoplankton community (Round et al., 1990; Hasle and Syvertsen, 1996). Based on a recent characterization of diatom diversity patterns on a global scale (Malviya et al., 2016), *Chaetoceros* and *Thalassiosira* represented the first and third most abundant ribotypes and were among the three most diverse genera. Although specific *Thalassiosira–*bacteria interactions have been studied (Gärdes et al., 2011; Durham et al., 2015; van Tol et al., 2017), the diversity of heterotrophic bacteria associated with both these globally significant phytoplankton genera is not well known. Furthermore, most past studies of the associations between phytoplankton and bacteria have used population or community-level approaches that may obscure cell-to-cell interactions. In this study, our major goal was to evaluate diatom-bacteria associations at an appropriate scale in focusing on the epibiotic microflora associated to *Chaetoceros* and *Thalassiosira* species. We evaluated the prevalence of attached bacteria to diatom cells and compared bacterial assemblages in both situations. We hypothesized that *in situ* bacterial associations differ from those in cultures and that the attachment of bacteria specific to each diatom was also favored in laboratory culture conditions.

## Materials and Methods

### Diatom Cultures and Natural Samples

Clonal strains of *T. delicatula* RCC 2560 and *Chaetoceros danicus* RCC 2565 were obtained from the Roscoff Culture Collection (RCC). Both diatoms have been isolated from the same sample of surface seawater (1 m depth) collected in January 2011 offshore Roscoff at the Astan observatory site (60 m depth, 48°46′40′′ N, 3°56′15′′ W) using the RV Neomysis. The long-term maintenance of both strains in the RCC since their isolation is performed by regular subculturing at intervals of 2 weeks. Both strains are grown at 19°C in K medium for diatoms (Keller et al., 1987) with a 14:10 h light:dark cycle at 80 μE.m^-2^.s^-1^.

Natural surface seawater samples (1 m depth) were collected at the Astan and Estacade (48°43′56′′ N, 3°58′58′′ W) sites in March/April 2014 (molecular approach) and in July/August 2014 (culture approach) to isolate *Thalassiosira* and *Chaetoceros* cells. A recent analysis of the microphytoplankton abundance and diversity at Astan demonstrated that *Thalassiosira* and *Chaetoceros* are abundant (above 500 cells.L^-1^ on average in all the 157 samples analyzed from 2000 to 2010) and able to become dominating at times (Guilloux et al., 2013). Diatom populations in this system vary throughout the year in both species diversity and abundance of individual species.

### Scanning Electron Microscopy

To visualize the epibionts attached on diatom cells, we roughly followed the protocol described by Kaczmarska et al. (2005). Diatom cultures (about 2 ml) were fixed for 2 h in 3% glutaraldehyde, filtered by gravity on 5 μm polycarbonate membranes (Isopore, Millipore) and washed three times with 10 ml of 0.22 μm filter-sterilized seawater. Samples were then dehydrated in a graded series of ethanol (two successive ethanol baths at 30, 50, 70, 90% and three at 100%) for at least 10 min at each grade. Ethanol solutions (3 ml) in the filtration tower were exchanged with gentle vacuum. After critical point drying (Bal-Ted CDP 030, Balzers, Liechtenstein), the samples were sputtered with gold and examined using a Phenom G2 Pro desktop scanning electron microscope (Phenom world). Only intact cells allowing visualization of one fully exposed side of the cingulum and valve or spine (according the diatom) at a time were analyzed. Only the bacteria that demonstrated clear evidence of attachment were counted (Kaczmarska et al., 2005).

### Single Diatom Cell Isolation

Single diatom cells were isolated under sterile conditions in a laminar flow hood. To lower the number of free-living bacteria in the algal cultures and to concentrate microalgae from natural seawater samples, algal cells were first gently separated by gravity using a 47 mm diameter, 11 μm pore-size nylon filter (Millipore) and washed three times with 50 mL of autoclaved seawater. Single cells were picked with a sterile glass capillary micropipette and washed 3–4 times with filter-sterilized seawater for further bacterial epibiont culture or with sterile phosphate-buffered saline (PBS; 37 mM NaCl, 2.7 mM KCl, 10 mM Na_2_HPO_4_, 2 mM KH_2_PO_4_, pH 7.5) for further direct molecular identification of epibionts. We previously observed that replacement of sterile seawater by PBS as washing solution improved the PCR amplification success. Single cells were directly transferred in culture medium or kept on ice until further DNA extraction. For both approaches, controls were performed for each diatom cell isolated by checking the absence of bacteria in the last seawater or PBS drop used in the washing series (see below). Since these isolation steps were time-consuming, several independent late exponential cultures of both diatoms and seawater samples were needed to isolate a sufficient number of epibionts using both approaches.

### Culture of Bacterial Epibionts

For cultivation of diatom epibionts, single isolated algal cells and controls were directly transferred in 48-well plates containing low-nutrient heterotrophic medium (LNHM) (Rappé et al., 2002) prepared by dissolving 35 g/L of commercial sea salts (Red Sea Europe) instead of using natural seawater. Bacterial cultures were incubated at 19°C for up to 6 weeks and growth was examined by flow cytometry using a BD Accuri C6 cytometer (BD Biosciences). For flow cytometry, 100 μl cultures were fixed with glutaraldehyde (0.25%, final concentration) and stained with SYBR Green (Life Technologies) as described by Marie et al. (1997). Cultures that contained bacteria were streaked on LNHM agar and selected colonies were purified by subculturing. Some of the isolates obtained in this study are available at the RCC.

### Dereplication of Bacterial Isolates

In order to eliminate duplicates, bacterial isolates were dereplicated by matrix-assisted laser desorption ionization–time of flight mass spectrometry (MALDI-TOF MS) (Ghyselinck et al., 2011). Colonies were obtained by growing the isolates on marine agar medium (1:10; 0.5 g peptone, 0.1 g yeast extract, 35 g sea salts dissolved in 1 L of Milli-Q water) at room temperature for 4–7 days according to their growth rate. A small amount of colony was directly applied onto a polished steel MSP 96 target plate (Bruker Daltonics). After drying, the deposited bacteria were overlaid with 1 μl of HCCA matrix (a saturated α-cyano-4-hydroxycinnamic acid in 50% acetonitrile and 2.5% trifluoroacetic acid; Bruker Daltonics) and air dried at room temperature. Mass spectra were acquired on a microflex LT MALDI-TOF mass spectrometer (Bruker Daltonics) configured with Bruker flexControl software using the default settings. Mass spectra were obtained in t2d format and were converted to txt files using the Data Explorer 4.9 software (AB Sciex). The txt files were imported in BioNumerics 5.1 software (Applied Maths) and converted to fingerprints for further analyses. To obtain reliable data analysis, the spectra with extensive noise and/or insufficient signal intensities were excluded. The similarity between the spectra was expressed using Pearson’s product moment correlation coefficient and the spectra were clustered using the UPGMA clustering algorithm.

### Molecular Analysis of Epibiotic Microflora

#### DNA Extraction From Single Cells and Whole-Genome Amplification (WGA)

DNA from single diatom cells and their attached bacteria was extracted following chemical treatment and thermal shock. Cells were lysed using lysis and neutralization buffers prepared as described in Humily et al. (2014). After addition of 0.5 μL of lysis buffer, the mixture was incubated at 4°C for 10 min in a thermocycler. The lysate was further incubated at 95°C for 1 min, cooled at 4°C before adding 0.5 μL of neutralization buffer, and kept 3 min on ice until WGA.

Whole-Genome Amplification reactions were carried out under a HEPA/UV3 PCR Workstation (UVP) using the Genomiphi v2 kit (GE Healthcare). WGA reactions were carried out in 12 μL final volume by adding sample buffer (3.5 μL), reaction buffer (4.5 μL), and phi29 enzyme (0.5 μL) and then incubated for 4 h at 30°C before inactivating the enzyme for 5 min at 65°C. Positive controls consisting of 1–9 bacterial cells in 3 μL of PBS were performed to check the efficiency of WGA reaction. Blank controls with sterile PBS were also performed for each experiment. WGA products were stored at -20°C until processing.

#### PCR Amplification and Sequencing of rRNA Genes

Whole-Genome Amplification products were tested for the presence of bacteria with primer 1492R (Turner et al., 1999) in combination with primer 799F (Chelius and Triplett, 2001; Ghyselinck et al., 2013) that strongly discriminates against chloroplast 16S rDNA. Reaction mixtures (12.5 μL) contained 0.75 U of GoTaq G2 Flexi DNA polymerase (Promega), 1X polymerase buffer, 2.0 mM MgCl_2_, 0.1 mM of each deoxynucleoside triphosphate, 0.2 μM of each primer, and 1 μL of WGA product. The program consisted of an initial denaturation step of 3 min at 95°C, followed by 35 cycles (20 s at 95°C, 40 s at 53°C, and 40 s at 72°C), and a final extension step of 10 min at 72°C. 16S rRNA genes from cultured epibionts were amplified with primers 8F (Turner et al., 1999) and 1492R using the conditions above. The program consisted of an initial denaturation step of 10 min at 94°C, followed by 35 cycles (30 s at 95°C, 1 min at 55°C, and 1 min at 72°C), and a final extension step of 10 min at 72°C.

In order to identify the diatom single cells isolated from the natural environment, the genes encoding their 18S rRNA gene and the large sub-unit (LSU) D1–D3 region were amplified using primers 63F and 1818R and D1R and D3Ca, respectively (Orsini et al., 2002). Reaction mixtures (15 μL) contained 0.75 U of GoTaq G2 Flexi DNA polymerase, 1X polymerase buffer, 2.0 mM MgCl_2_, 0.2 μM of each primer, 0.1 mM of each deoxynucleoside triphosphate, and 1 μL of extracted DNA. The program consisted of an initial denaturation step of 5 min at 95°C, followed by 40 cycles (30 s at 95°C, 30 s at 50°C (for 18S rRNA amplification) or 30 s at 55°C (for LSU amplification), and 1 min at 72°C) and a final extension step of 10 min at 72°C.

Whole-Genome Amplification products that proved positive for 16S rRNA but negative for 18S rRNA were removed from further analysis. PCR products of 16S rRNA genes were cloned using TOPO TA Cloning Kit^®^ (Invitrogen) as recommended by the manufacturer. Insert-containing clones were identified by agarose gel electrophoresis of PCR products amplified using M13F and M13R primers. Clones and PCR products were sequenced by Macrogen Europe (Amsterdam, Netherlands) or by the Biogenouest sequencing platform at the Station Biologique (Roscoff, France). Bacterial taxon for each sequence was identified and named by the homologous 16S sequence in Genbank using BLAST (Altschul et al., 1990). Phylogenetic analyses of 16S and 18S rRNA gene sequences were performed using the neighbor joining tree method implemented in MEGA6 software (Tamura et al., 2013).

### Statistical Analyses of the Bacterial Communities

Each single algal cell was considered as an environment to which 16S rDNA sequences were assigned. We used the unweighted UniFrac distance measure (Lozupone and Knight, 2005; Hamady et al., 2010) to compare the presence or absence of taxa with the bacterial communities. To determine whether the cultured and natural bacterial communities in both diatoms were significantly different than random, we used the Unifrac significance test along with the principal coordinates analysis (PCoA) both run in Mothur (Schloss et al., 2009).

### Nucleotide Sequence Accession Numbers

All nucleotide sequences obtained in this study are available in GenBank database under the accession numbers KX197296 to KX197383, and KU926270 (16S rRNA of cultured bacteria), KX197247 to KX197295 (16S rRNA of uncultured bacteria), and KX226392 to KX226398 (16S rRNA and LSU region of microalgae).

## Results and Discussion

### Bacterial Colonization of Diatom Cells

In this study, we first compare the prevalence of bacterial cell attachment in both diatom species. It is well known that bacterial colonization may be influenced by the algal growth state in cultures and the bloom stage in natural samples (Grossart et al., 2005; Kaczmarska et al., 2005). To circumvent this issue, algal cultures used for single cell isolation were all in early stationary growth phase and the same natural samples were used to isolate *Chaetoceros* and *Thalassiosira* cells. A total of 296 and 469 cells were manually isolated from independent cultures and natural samples, respectively (Table 1). Both culture-based and molecular approaches yielded generally nearly identical proportions of *Chaetoceros* cells colonized by bacteria. However, both methods yielded different epibiont proportions in *Thalassiosira.* This was somewhat surprising because (i) the observed epibiont proportions were remarkably stable in the different *Thalassiosira* RCC 2560 cultures used for epibiont isolation whatever the approach used (see the section “Materials and Methods”) and (ii) the high epibiont prevalences observed using the culture-based approach were consistent with that measured by scanning electron microsopy (Supplementary Table 1). From these results, a reasonable explanation is that the molecular approach is most prone to yield variable results. Epibiont proportions in diatom cells isolated from the environment were generally lower than that from the cultures as measured using the culture-based approach. This result makes sense because we observed a majority of diatom cells free of epibiotic bacteria (Table 1), with proportions exceeding 90% for *Chaetoceros* in natural waters. These observations are in agreement with early studies that led to the conclusion that living pelagic diatoms are not colonized by bacteria (Droop and Elson, 1966) and with more recent reports that showed high proportions of bacteria-free diatom cells in old cultures (Kaczmarska et al., 2005). They also support the hypothesis that the extent of algal colonization by bacteria are positively related to the densities of both free bacteria and microalgae (Vaqué et al., 1989). This may also explain the differential proportions of diatom cells colonized by bacteria we found in cultures and in natural samples. However, we can also assume that cells in culture are confined to uniform laboratory conditions favoring stable interactions between partners and colonization while a range of environmental factors can positively or negatively influence temporary algal–bacterial relationships in natural conditions.

**Table 1:** Proportions of algal cells with bacterial epibionts in cultures and natural samples as evaluated by culture-based and molecular approaches and numbers of isolates and bacterial sequences obtained in this study.

	Cultures		Natural samples	
	*Chaetoceros* RCC 2565	*Thalassiosira* RCC 2560	*Chaetoceros* spp.	*Thalassiosira* spp.
***Culture approach***				
Algal cells isolated^a^	75	88	206	209
Algal cells with epibionts^b^ (%)	27 (36)	69 (78)	19 (9)	68 (33)
Epibiont isolates^c^	38	63	12	64
Bacterial species^d^	5	14	7	38
***Molecular approach***				
Algal cells isolated^e^	20	113	28	26
Algal cells with epibionts^f^ (%)	6 (30)	38 (34)	2 (7)	14 (54)
PCR libraries from positive WGAs	6	18	2	14
Bacterial phylotypes	9	19	3	14
Bacterial species^d^	7	4	3	10

*^a^Cells for which no bacterial growth was obtained from controls. ^b^Positive enrichments in LNHM as measured by flow cytometry. ^c^Epibionts isolated on LNHM agar plates. ^d^Defined at the 98% similarity level. ^e^Cells for which no amplification products were obtained from controls. ^f^Proportions of positive WGAs with negative controls.*

Only limited quantitative information exists on the interactions between attached bacteria and phytoplankton in pelagic aquatic environments. However, the actual view is that bacterial colonization of planktonic algae may vary with respect to algal species and physiological state (Grossart et al., 2005, 2006). Indeed, we found that *Thalassiosira* cells harbored significantly higher proportions of epibionts than *Chaetoceros* cells whatever the approach used (Table 1) and this was confirmed by SEM data (Supplementary Table 1). Two possibilities can bring out the difference observed. Since associations between bacteria and microalgae are known to change over the course of algal bloom cycles (Grossart et al., 2005, 2006; Mayali et al., 2011), one possibility is that the effect of temporal variation in algal hosts collections. However, it is not the case in our samples, because *Thalassiosira* and *Chaetoceros* cells were isolated from the same natural samples although there was a 4–5 month time difference between cell collections for both approaches. Since the epibiont proportions obtained by both approaches for each diatom showed the same trends, it is unlikely that the differences observed are linked to different physiological states. A likely possibility is that diatom hosts are quite distinct in their size and structure characteristics, their release of exopolymers and their production of inhibitory substances (Vaqué et al., 1989; Myklestad, 1995; Amin et al., 2012).

Using the cultivation and molecular data, we calculated the average number of species or OTUs attached to single diatoms cells. Interestingly, whatever the approach used, we found that each host cell harbored between 1 to 2.3 epibiotic species or OTUs, suggesting a rather low attachment. This average number is a minimum estimate of the number of bacterial cells that occurred on the host cell, but with the methods used it was not possible to evaluate how many bacterial cells were attached to the host cells examined. However, these numbers are close to that we obtained using SEM on late exponential cultures (0.8–3.8 bacterial epibionts on cingulum or spines/valve; Supplementary Table 1). These low numbers were generally in agreement with that reported by Baker and Kemp (2014) although these authors found also higher numbers of phylotypes (up to 11) per algal cell for some species or strains.

### Microbial Community Comparisons Between Hosts in Short- and Long-Term Associations

Using the molecular method, a total of 45 bacterial phylotypes were obtained after WGA of 40 single diatom cells isolated from collection cultures and natural samples (Tables 1, 2). When clustered at the 98% similarity level, 24 OTUs representing diverse lineages of bacterial phyla were identified and their distribution did not overlap among host species and between collection cultures and environmental samples. The identified OTUs were classified into the classes *Alpha*-, *Beta*- and *Gammaproteobacteria, Actinobacteria, Flavobacteriia, Bacilli, Cytophagia*, and *Sphingobacteria*. Bacterial cultivation using a low-nutrient organic medium and extended incubation periods of up to 6 weeks were used to increase the overall assessment of the species richness. Cultivation identified a total of 177 unique isolates (Tables 1, 3, 4). The isolated strains fell generally into the same bacterial classes than environmental clones but none belonged to the classes *Betaproteobacteria* and *Bacilli*.

**FIGURE 1:**
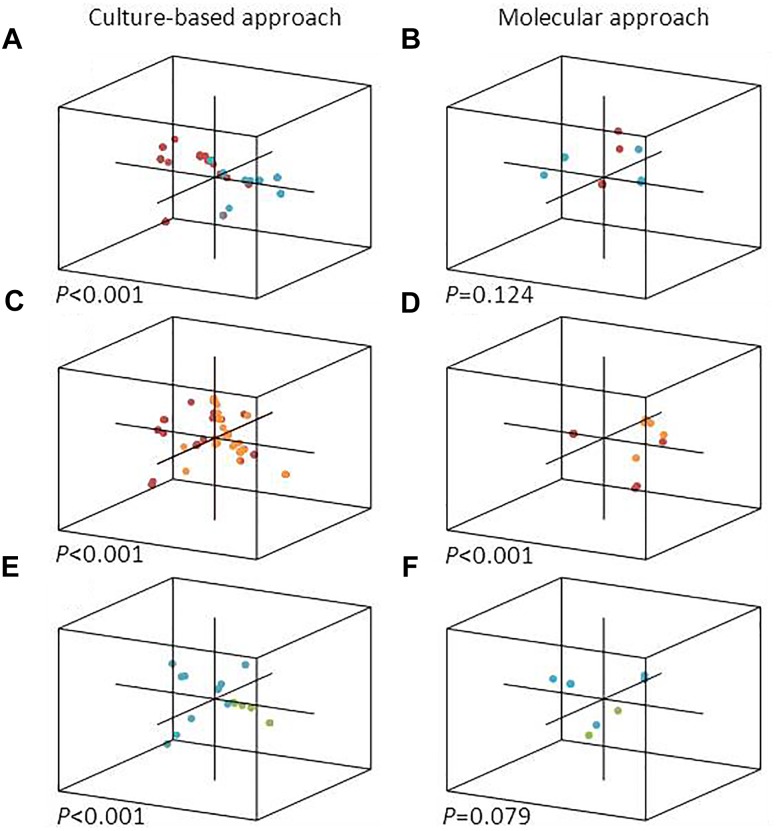
PCoA representation of each single diatom cell analyzed in this study, with a 3D position depending on the composition of its epibiotic bacterial community. Culture-based (left) and molecular (right) bacterial diversity attached to diatoms isolated from collection cultures **(A,B)**, natural samples **(E,F)** and both **(C,D)** are shown. Diatom cells correspond to *T. delicatula* RCC 2560 (red), *C. danicus* RCC 2565 (blue), environmental *Thalassiosira* spp. (orange), and environmental *Chaetoceros* spp. (green).

In this study, we asked if microalgal species could influence the epibiont community structure in cultures and in natural communities. Unweighted UniFrac analyses, which takes into account only presence/absence data for OTUs/species, showed that the epibiotic bacterial communities associated with *T. delicatula* RCC 2560 and *C. danicus* RCC 2565 were significantly distinct (*P* < 0.001) as determined by the culture-based approach. Principal Coordinate Analysis (PCoA) also separated the corresponding libraries (Figure 1A). No significant dissimilarities were shown between epibiont communities of the cultures as determined by the molecular approach (*P* = 0.124) (Figure 1B). Our results complement, however, earlier observations suggesting that microalgal cultures harbor specific bacterial communities (Schäfer et al., 2002; Grossart et al., 2005; Sapp et al., 2007; Guannel et al., 2011; Behringer et al., 2018). We also compared the culture-based and molecular bacterial diversity attached to *Thalassiosira* and *Chaetoceros* single cells isolated from natural samples and collection cultures, considered here as short- and long-term associations, respectively. Both methods indicated that epibiotic bacterial communities associated with cultures and environmental cells of *Thalassiosira* showed a highly significant difference (*P* < 0.001) (Figures 1C,D). Epibiont assemblages in cultured and environmental *Chaetoceros* cells also differed significantly as determined by culture-based approach (*P* < 0.001) while molecular libraries showed only marginally significant difference (*P* = 0.079) (Figures 1E,F). Since single cells isolated from natural waters belonged to two *Chateoceros* species, we enlarged the specificity of *in situ* associations to the genus level. Nevertheless, distinctness between *in situ* epibiotic communities of both diatom genera was observed in the same samples collected at the same place. This result is in line with previous findings showing that bacterial assemblages associated with phytoplankton cultures can be very different from the natural bacterial assemblages during blooms of the same species (Garcés et al., 2007). We acknowledge, however, that the diatom hosts isolated from natural waters differed at the species level from their cultured relatives.

### Diversity of Diatom Epibionts

Although the molecular approach was designed and performed using necessary precautions (decontamination and cleaning procedures) to prevent contamination, the successive steps that include single cell isolation, whole genome and PCR amplification presented opportunities for the inclusion of non-host derived bacterial sequences. We also removed samples with any negative control amplification from further analysis. We didn’t find sequences recognized as being commonly associated to human skin but a few phylotypes and strains were not typical of bacterial taxa found previously with algal cultures (Tables 2–4) Among them, we identified sequences of *Aeribacillus pallidus* (*Firmicutes*) that were abundantly found in skin-associated bacterial communities of marine fishes (Larsen et al., 2013). Like other Gram-positive bacteria frequently found in marine microalgal laboratory cultures, we suspect their presence is due to a contamination from handling (Nicolas et al., 2004). Although *Actinobacteria* are known to be rare in pelagic marine environments (Pommier et al., 2007), we obtained several actinobacterial strains and sequences attached to diatom cells in cultures and natural samples (Tables 2–4). Interestingly, the isolates belong to genera with species obtained from diatom cultures (Le Chevanton et al., 2013) and from marine environments, most of them in association with invertebrates, algae and microalgae (Green et al., 2004; Pathom-aree et al., 2006; Menezes et al., 2010; Palomo et al., 2013). We therefore cannot exclude that these bacteria represent rare members in the diatom phycosphere.

**Table 2:** Epibiotic bacterial phylotypes recovered from diatom cells in culture and from natural waters and their closest cultivated relative identified by Blastn.

Diatom host	*n*^a^	Phylum/class	Order	Family	Species	16S rRNA similarity (%)
**Cultures**						
*C. danicus* RCC 2565	3	*Alphaproteobacteria*	*Rhodobacterales*	*Hyphomonadaceae*	*Algimonas* sp.	99.6
	1	*Betaproteobacteria*	*Burkholderiales*	*Burkholderiaceae*	*Limnobacter thiooxidans*	100
	1			*Comamonadaceae*	*Pelomonas* sp.	99.9
	1	*Gammaproteobacteria*	*Oceanospirillales*	*Halomonadaceae*	*Halomonas* sp	99.9
	1	*Flavobacteriia*	*Flavobacteriales*	*Cryomorphaceae*	*Brumimicrobium mesophilum*	93.5
	1	*Actinobacteria*	*Micrococcales*	*Micrococcaceae*	*Nesterenkonia flava*	99.4
	1	*Bacilli*	*Bacillales*	*Bacillaceae*	*Aeribacillus pallidus*	99.9
*T. delicatula* RCC 2560	1	*Alphaproteobacteria*	*Sphingomonadales*	*Erythrobacteraceae*	*Erythrobacter litoralis*	98.8
	1		*Rhodobacterales*	*Rhodobacteraceae*	*Maribius pelagius*	94.7
	1	*Bacilli*	*Bacillales*	*Bacillaceae*	*Aeribacillus pallidus*	100
	16	*Flavobacteriia*	*Flavobacteriales*	*Flavobacteriaceae*	*Tenacibaculum skagerrakense*	97.8
**Environmental cells^b^**				
*Chaetoceros debilis*	1	*Alphaproteobacteria*	*Rhodobacterales*	*Rhodobacteraceae*	*Marivita cryptomonadis*	98.5
	1				*Pseudoruegeria lutimaris*	97
*Chaetoceros protuberans*	1	*Gammaproteobacteria*	*Alteromonadales*	*Alteromonadaceae*	*Saccharophagus degradans*	95.1
*Thalassiosira punctigera*	1	*Alphaproteobacteria*	*Rhodobacterales*	*Rhodobacteraceae*	*Roseovarius nubinhibens*	97.9
	1	*Gammaproteobacteria*	*Alteromonadales*	*Colwelliaceae*	*Colwellia psychroerythraea*	99.3
	1		*Chromatiales*	*Ectothiorhodospiraceae*	*Ectothiorhodospira mobilis*	94.3
	1		*Cellvibrionales*	*Halieaceae*	*Chromatocurvus halotolerans*	95.7
	1			*Spongiibacteraceae*	*Dasania marina*	93
	1				*Oceanicoccus sagamiensis*	96.8
	1		*Pseudomonadales*	*Pseudomonadaceae*	*Pseudomonas trivialis*	90.7
	4	*Cytophagia*	*Cytophagales*	*Amoebophilaceae*	*Candidatus Amoebophilus asiaticus*	93
	2	*Flavobacteriia*	*Flavobacteriales*	*Cryomorphaceae*	*Salinirepens amamiensis*	94.4
	1	*Sphingobacteriia*	*Sphingobacteriales*	*Saprospiraceae*	*Lewinella cohaerens*	89.1

*^a^Number of individual diatom cell carrying the corresponding bacterial phylotype; ^b^as identified by 18S rRNA sequencing.*

Epibiotic bacteria isolated from natural diatom populations were highly diverse (Table 3) and only a few species overlapped in *Chaetoceros* and *Thalassiosira* cells. Interestingly, the overlapping bacterial species were also the most frequently isolated from both genera. They consisted in species of the genera *Pseudoalteromonas* and *Tenacibaculum*, the majority of which appear to be associated with eukaryotic hosts, including various marine algae (Akagawa-Matsushita et al., 1992; Egan et al., 2000, 2001; Suzuki et al., 2001). Strains and phylotypes of *Tenacibaculum* were also repeatedly isolated and amplified from cells of *T. delicatula* RCC 2560, suggesting a possible physiological adaptation to an epiphytic lifestyle. This hypothesis is in line with the ecology of *Tenacibaculum* species known to be often associated with surfaces of marine invertebrates, fishes and microalgae and responsible of skin lesions and algicidal activity (Piñeiro-Vidal et al., 2012; Li et al., 2013).

**Table 3:** Epibiotic bacterial strains isolated from diatom cells in natural waters and their closest cultivated relative identified by Blastn.

Diatom host	Strain (n)	Phylum/class	Order	Family	Species	16S rRNA similarity (%)
*Chaetoceros* spp.	KC58 (2)	*Alphaproteobacteria*	*Rhizobiales*	*Phyllobacteriaceae*	*Hoeflea phototrophica*	97.9
	KC43 (1)	*Gammaproteobacteria*	*Alteromonadales*	*Alteromonadaceae*	*Glaciecola punicea*	96
	KC55B (4)			*Pseudoalteromonadaceae*	*Pseudoalteromonas undina/marina*	99
	KC57 (1)				*Pseudoalteromonas carrageenovora*	100
	KC61 (1)		*Vibrionales*	*Vibrionaceae*	*Photobacterium aquimaris*	99.9
	KC46A (1)	*Flavobacteriia*	*Flavobacteriales*	*Flavobacteriaceae*	*Tenacibaculum gallaicum*	97.7
	KC79A (2)	*Actinobacteria*	*Actinomycetales*	*Micrococcaceae*	*Arthrobacter subterraneus*	99.8
*Thalassiosira* spp.	KC76 (1)	*Alphaproteobacteria*	*Kiloniellales*	*Kiloniellaceae*	*Kiloniella laminariae*	96.3
	KC56 (1)		*Rhizobiales*	*Hyphomicrobiaceae*	*Hyphomicrobium nitrativorans*	95.3
	KC41B (1)			*Phyllobacteriaceae*	*Nitratireductor indicus*	95.2
	KC41A (1)				*Nitratireductor pacificus*	95.2
	KC45 (1)		*Rhodobacterales*	*Rhodobacteraceae*	*Jannaschia sp.*	98
	KC59 (1)				*Litoreibacter meonggei*	97.7
	KC77A (2)				*Loktanella maricola*	99.6
	KC68 (1)				*Loktanella vestfoldensis*	96
	KC75 (1)				*Nereida ignava*	97.3
	KC63A (3)				*Octadecabacter antarcticus*	98.1
	KC47 (1)				*Roseobacter litoralis*	98.8
	KC52A (2)				*Shimia marina*	98.2
	KC42A (2)		*Sphingomonadales*	*Erythrobacteraceae*	*Altererythrobacter luteolus*	97
	KC74 (1)				*Croceicoccus marinus*	97.3
	KC49 (1)				*Erythrobacter aquimaris*	99.6
	KC67 (1)			*Sphingomonadaceae*	*Sphingopyxis flavimaris*	98.8
	KC60 (1)	*Cytophagia*	*Cytophagales*	*Flammeovirgaceae*	*Roseivirga ehrenbergii*	93.2
	KC65A (2)	*Flavobacteriia*	*Flavobacteriales*	*Flavobacteriaceae*	*Croceitalea eckloniae*	97.3
	KC50A (2)				*Dokdonia genica*	99.7
	KC62 (1)				*Lacinutrix sp.*	97.3
	KC44 (1)				*Lutimonas vermicola*	100
	KC51B (2)				*Maribacter aquivivus*	97.7
	KC51A (1)				*Maribacter forsetii*	96
	KC81 (1)				*Maribacter ulvicola*	99.3
	KC73A (2)				*Olleya namhaensis*	97.3
	KC46B (2)				*Tenacibaculum gallaicum*	97.7
	KC48 (1)	*Gammaproteobacteria*	*Alteromonadales*	*Alteromonadaceae*	*Aestuariibacter litoralis*	97.8
	KC78 (1)				*Alteromonas tagae*	99
	KC72 (1)				*Pseudohaliea rubra*	98
	KC53A (5)			*Pseudoalteromonadaceae*	*Pseudoalteromonas undina/marina*	99.9
	KC66 (1)			*Shewanellaceae*	*Shewanella japonica*	99.3
	KC70 (1)			*Colwelliaceae*	*Thalassomonas agariperforans*	96.9
	KC64 (1)		*Chromatiales*	*Granulosicoccaceae*	*Granulosicoccus coccoides*	96.9
	KC80 (1)		*Vibrionales*	*Vibrionaceae*	*Vibrio breoganii*	100
	KC54 (1)				*Vibrio splendidus*	100
	KC71 (1)		Unclassified		*Chromatocurvus halotolerans*	97.3
			*Gammaproteobacteria*
	KC69 (1)	*Sphingobacteriia*	*Sphingobacteriales*	*Saprospiraceae*	*Portibacter lacus*	91.5
	KC79C (1)	*Actinobacteria*	*Actinomycetales*	*Micrococcaceae*	*Arthrobacter subterraneus*	99.8

*^a^Number of individual diatom cell carrying the corresponding bacterial isolate; ^b^based on partial sequences >663 bp.*

*Alpha*- and *Gammaproteobacteria* were the two main classes identified in the cultivable epibiotic communities associated with cultures (Table 4 and Supplementary Figure 1). Interestingly, some epibionts were regularly isolated from both diatom cultures. Among them, strains belonging to the gammaproteobacterial genus *Marinobacter* are common inhabitants of phytoplankton cultures (Amin et al., 2009, 2015; Le Chevanton et al., 2013; Sison-Mangus et al., 2014; Green et al., 2015; Lupette et al., 2016). While investigating the bacterial community associated with dinoflagellates and coccolithophores, Amin et al. (2009) observed that members of the genus *Marinobacter* were present in over 80% of cultures. Further studies demonstrated that *Marinobacter* isolates formed specific beneficial associations with diverse phytoplankton that could require cell-to-cell adhesion (Amin et al., 2009; Bolch et al., 2011; Gärdes et al., 2011). Other frequently isolated epibiotic bacteria were affiliated with the *Rhodobacterales* and they differed with the algal species. *Rhodobacterales* appear also to be particularly well-adapted to close association with phytoplankton in general and have been shown to increase during phytoplankton blooms (Mayali et al., 2011; Teeling et al., 2012). Laboratory experiments involving specific co-culture experiments also indicate that widespread interactions may occur between phytoplankton and Roseobacters (Durham et al., 2015; Segev et al., 2016; Seymour et al., 2017). Cultivated attached microflora of *C. danicus* RCC 2565 were related to the genera *Algimonas* while *Erythrobacter, Paracoccus* and a new genus within the family *Rhodobacteraceae*, recently described as *Silicimonas algicola* (Crenn et al., 2016), prevailed in *T. delicatula* RCC 2560. *Algimonas, Erythrobacter*, and *Paracoccus* are also reported for their common occurrence in algal cultures or as epibionts of macroalgae (Alavi et al., 2001; Schäfer et al., 2002; Jasti et al., 2005; Kaczmarska et al., 2005; Le Chevanton et al., 2013; Cruz-López and Maske, 2016).

**Table 4:** Epibiotic bacterial strains isolated from diatom cells in culture and their closest cultivated relative identified by Blastn.

Diatom host	Strain (n)	Phylum/class	Order	Family	Species	16S rRNA similarity (%)
*C. danicus* RCC 2565	KC05A (17)	*Alphaproteobacteria*	*Caulobacterales*	*Hyphomonadaceae*	*Algimonas ampicilliniresistens*	98
	KC39 (2)		*Rhodobacterales*	*Rhodobacteraceae*	*Sulfitobacter dubius*	99.9
	KC25 (11)	*Gammaproteobacteria*	*Alteromonadales*	*Alteromonadaceae*	*Marinobacter sediminum*	99.8
	KC36 (1)				*Marinobacter lipolyticus*	99.8
	KC86 (1)				*Silicimonas algicola*	100
*T. delicatula* RCC 2560	KC04 (1)	*Alphaproteobacteria*	*Sphingomonadales*	*Erythrobacteraceae*	*Altererythrobacter ishigakiensis*	97.9
	KC12 (26)				*Erythrobacter gaetbuli*	98
	KC18 (1)				*Erythrobacter citreus*	98.3
	KC90 (15)		*Rhodobacterales*	*Rhodobacteraceae*	*Silicimonas algicola*	100
	KC10 (10)				*Paracoccus aminophilus*	96.7
	KC17B (1)				*Paracoccus stylophorae*	97.8
	KC38C (1)KC16 (3)	*Gammaproteobacteria*	*Alteromonadales*	*Alteromonadaceae*	*Sulfitobacter dubiusMarinobacter salarius*	99.999.9
	KC31 (3)				*Marinobacter sediminum*	100
	KC14 (3)	*Flavobacteriia*	*Flavobacteriales*	*Flavobacteriaceae*	*Tenacibaculum sp*	95
	KC02 (1)	*Actinobacteria*	*Actinomycetales*	*Micrococcaceae*	*Kocuria rosea*	99.7
	KC23 (1)				*Micrococcus yunnanensis*	99.4
	KC03 (1)			*Nocardioidaceae*	*Nocardioides furvisabuli*	99.6
	KC24 (1)			*Dermacoccaceae*	*Dermacoccus nishinomiyaensis*	98.8

*^a^Number of individual diatom cell carrying the corresponding bacterial isolate; ^b^based on partial sequences >663 bp.*

Phylotypes recovered from diatom cells in culture and from natural waters were diverse and only a few of them were repeatedly obtained (Table 2). Similarly to *Tenacibaculum* species that were regularly amplified and isolated from cells of *T. delicatula* RCC 2560, sequences and strains of *Algimonas* were often obtained from cells of *C. danicus* RCC 2565, confirming the presence of members of this genus in *Chaetoceros* cultures (Le Chevanton et al., 2013). A phylotype distantly related to the obligate amoeba endosymbiont “*Candidatus* Amoebophilus asiaticus” was retrieved from environmental cells of *T. punctigera*. Although it was repeatedly amplified from single cells, it is impossible to draw any hypothesis on the possible relationships between these partners in natural waters.

A total of 56 unique isolates were obtained in this study (Tables 3, 4). We used the 16S rRNA gene identity cut-off value of 98.7% to define isolates that may represent novel species (Stackebrandt and Ebers, 2006). Overall, 35 unique isolates (>60% of the total isolates) showed 16S rRNA gene identity below this threshold and therefore may be potential candidates of new taxa. Of the new taxa, 5 isolates may represent novel genera at the conservative 95–96% identity cut-off (Yarza et al., 2014). Although 16S rRNA gene identities are based on partial sequences and our study does not prove that these isolates represent novel species, it provides a framework for isolating large numbers of epibiotic bacteria for possible novel taxa that may be of ecological importance. Indeed, repeated isolation of particular bacterial species from microalgae could be indicative of niche specificity or even established mutualistic relationships (Amin et al., 2009, 2012).

Our data demonstrated that diversity of the cultured epibiotic microflora was lower in diatom culture than in natural waters. Furthermore, the most frequently isolated taxa from diatom cultures were unusually isolated or amplified from environmental cells (Tables 2, 3). This is exemplified by *Marinobacter* species that we regularly grown from diatoms originally isolated from Roscoff coastal waters but were not found as diatom epibionts in natural waters off Roscoff, confirming the typically low annual relative abundance of these bacteria in the Western English Channel (Green et al., 2015). Together, our results provide some support to the hypothesis that, in uniform laboratory culture conditions, bacteria–bacteria and bacteria–diatom interactions selected for a simplified, but specific, epibiotic microbiota shaped and adapted for long-term associations. Since recent observations showed that cultivation in the laboratory for longer than 1 year resulted in only small changes in the bacteria composition suggesting robust associations between diatoms and their associated bacterial communities (Behringer et al., 2018), further work is needed to how these interactions can differ across algal species. Our findings reinforce also previous reports that showed that the amount and composition of the organic matter released by phytoplankton like polysaccharides, small amino acids, sugars, proteoglycans or glycoproteins, may act as selective agents for bacterial types (Myklestad, 1995; Sapp et al., 2007; Sarmento et al., 2013). We assume that bacterial groups able to develop an algal-attached lifestyle are probably more affected by this selection process.

### Methodological Considerations

This study addressed the examination of interactions between attached bacteria and two environmentally relevant diatom genera. Most studies that tried to tackle specifically this question first separated free-living bacteria from diatom cells by filtration on membranes and examined the bacterial assemblages associated to the diatom cell fraction (Kaczmarska et al., 2005; Mayali et al., 2011). We assume that the issue of specific associations cannot be answered using this method because non-attached bacteria may also be retained by membranes, and remain on them even after extensive washing steps. The methods described in our study and that employed by Baker and Kemp (2014) ensure the epibiotic status of the bacterial communities analyzed. The main limitation of our approach is that the manual isolation of single cells is time-consuming and limits the number of analyzed cells. Future studies might apply different strategies to pursue this question further. Although they require an expensive and sophisticated equipment and special infrastructure such as a clean room (Baker and Kemp, 2014), flow cytometry cell sorting systems may represent powerful tools to facilitate the rapid and efficient isolation of microalgae. They could greatly improve the analysis of attached bacterial assemblages in multiple cultures and environmental cells.

## Conclusion

Our observations complement previous studies which addressed the existence of algal-specific bacterial communities. The present analysis of the microflora attached to ubiquitous marine diatoms demonstrated conclusively that abundance and community composition of epibiotic bacteria may vary significantly with algal species. The dominance of certain epibiotic bacteria, either common or specific to algal species, together with the simplification of bacterial communities along regular algal subculturing indicate selection of bacteria highly adapted to long-term interactions with hosts.

In the context of finding bacteria that could have symbiotic interactions with diatoms, the bacterial strains we repeatedly isolated from cultures and environmental cells represent good candidates. Further co-cultures experiments with axenic cultures of *T. delicatula* RCC 2560 and *C. danicus* RCC 2565 evaluating the effect of bacteria on both microalgae could help us to determine the functional role of specific isolates.

## Author Contributions

KC designed and performed the experiments (diversity analysis) and wrote the manuscript. DD performed the experiments (diversity analysis). CJ designed the experiments and wrote the manuscript.

## Conflict of Interest Statement

The authors declare that the research was conducted in the absence of any commercial or financial relationships that could be construed as a potential conflict of interest.

## Supplementary Material

FIGURE S1 Maximum-likelihood tree based on 16S rRNA gene sequences showing the position of bacterial epibionts isolated from *C. danicus* RCC 2565 and *T. delicatula* RCC 2560 (Table 4). Only bootstrap values (expressed as percentages of 1000 replications) of >80% are shown. Filled circles indicate that the corresponding nodes were also recovered using the neighbor-joining algorithm. *Thermococcus litoralis* JCM8560^T^ was used as outgroup. Bar, 0.05 substitutions per nucleotide position.

TABLE S1 Attached bacteria on diatom cultures during late exponential (5 days) and late stationary (19 days) growth phases.
