# Supplementary figures and images for: Bacterial Epibiotic Communities of Ubiquitous and Abundant Marine Diatoms Are Distinct in Short- and Long-Term Associations
Source: Front Microbiol. 2018 Dec 4;9:2879. doi: 10.3389/fmicb.2018.02879 (PMC6288172; doi:10.3389/fmicb.2018.02879)

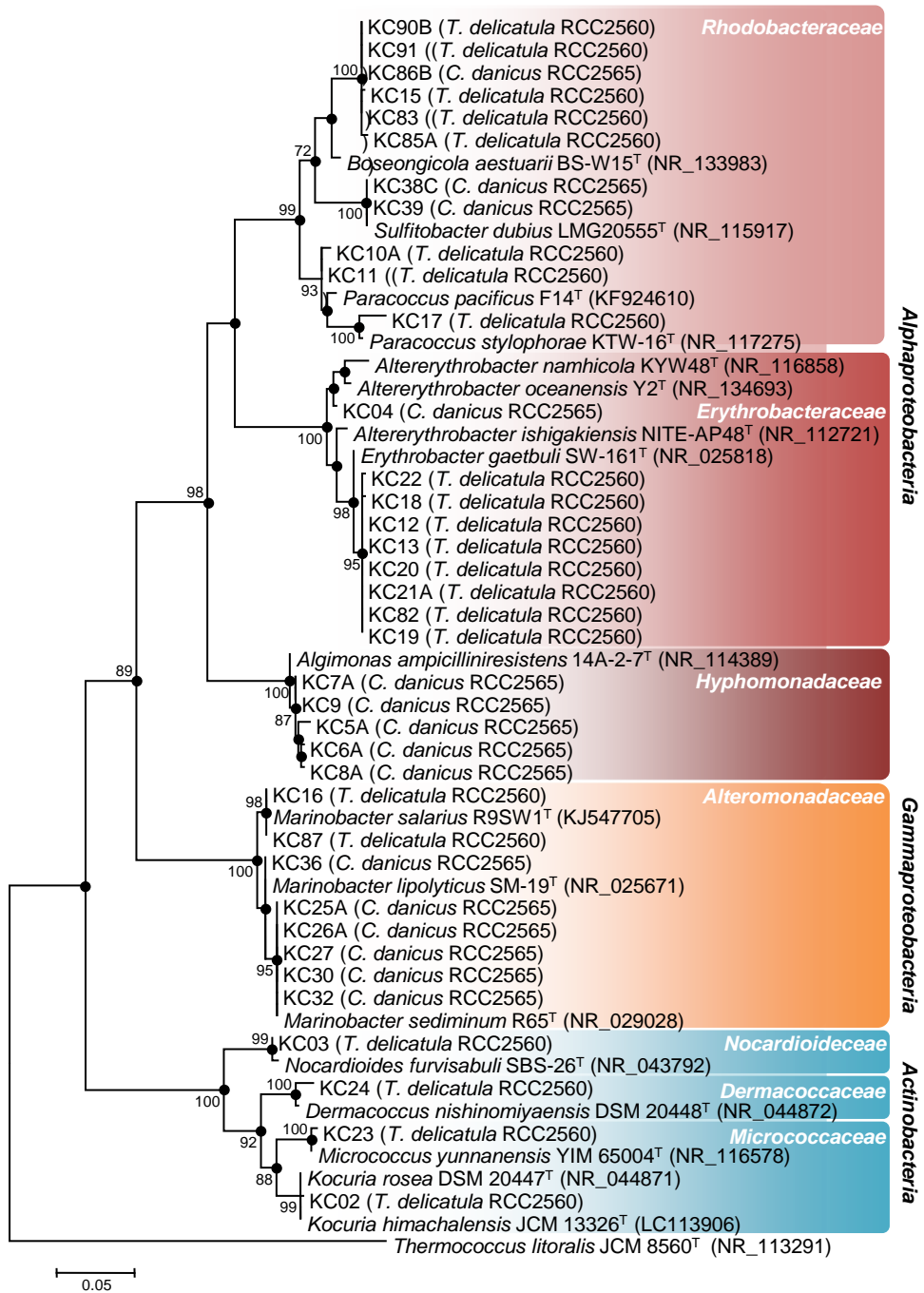

Crenn et al. Supplementary Fig 1

Supplement: FIGURE S1 — Maximum-likelihood tree based on 16S rRNA gene sequences showing the position of bacterial epibionts isolated from C. danicus RCC 2565 and T. delicatula RCC 2560 (Table 4). Only bootstrap values (expressed as percentages of 1000 replications) of >80% are shown. Filled circles indicate that the corresponding nodes were also recovered using the neighbor-joining algorithm. Thermococcus litoralis JCM8560T was used as outgroup. Bar, 0.05 substitutions per nucleotide position. [file Data_Sheet_1.PDF]
