# Supplementary material for: Bacterial Epibiotic Communities of Ubiquitous and Abundant Marine Diatoms Are Distinct in Short- and Long-Term Associations
Source: Front Microbiol. 2018 Dec 4;9:2879. doi: 10.3389/fmicb.2018.02879 (PMC6288172; doi:10.3389/fmicb.2018.02879)
Supplement: TABLE S1 — Attached bacteria on diatom cultures during late exponential (5 days) and late stationary (19 days) growth phases. [file Table_1.PDF]

Supplementary Table 1. Attached bacteria on diatom cultures during late exponential (5 days) and late stationary (19 days) growth phases

	C. danicus RCC 2565		*T. delicatula* RCC 2560	
	5 days	19 days	5 days	19 days
Algal cells with bacterial epibionts on cingulum (%)	63	38	83	97
Average number of bacterial epibionts on cingulum	0.8	0.65	3.8	6.4
Algal cells with bacterial epibionts on spines or valve[a] (%)	46	79	80	100
Average number of bacterial epibionts on spines or valve	0.8	1.7	2.8	6.5

[a]spines for *C. danicus* and valve for *T. delicatula*
